# Regulation of sepsis-induced IFNγ upon natural killer cell or natural killer T cell depletion *in vivo*

**DOI:** 10.1186/cc11733

**Published:** 2012-11-14

**Authors:** E Christaki, E Diza, EJ Giamarellos-Bourboulis, N Papadopoulou, A Pistiki, D Droggiti, A Machova, M Georgitsi, D Lambrelli, G Karkavelas, A Iliadis, N Malisiovas, P Nikolaidis, SM Opal

**Affiliations:** 1 Aristotle University of Thessaloniki Medical School, Thessaloniki, Greece; 2 University of Athens Medical School, Athens, Greece; 3 University of Cologne, Institute for Medical Microbiology Immunology and Hygiene, Cologne, Germany; 4 University of Macedonia, Thessaloniki, Greece; 5 Alpert School of Medicine of Brown University, Providence, RI, USA

## Background

Natural killer (NK) and natural killer T (NKT) cells play a key role in bacterial infection and sepsis since they contribute to the bridging of innate and acquired immune responses. We have previously shown that *in vivo *depletion of these cell populations in a murine pneumococcal pneumonia sepsis model affected mortality.

## Methods

Four groups of C57BL/6 mice (*n *= 5 to 15 mice/group) were infected intratracheally with 5 × 10^5 ^CFU *Streptococcus pneumoniae*. Twenty-four hours prior to bacterial inoculation, NK cell depletion was achieved by intravenous (i.v.) administration of anti-asialoGM1 rabbit polyclonal antibody in one group (NK^DEPL^), or anti-CD1d monoclonal antibody, clone 1B1 was given for NKT cell depletion in a second group (NKT^DEPL^). The control group received equal volume of isotype antibody control i.v. (C) and a fourth group received sham intratracheal installation of normal saline (S). All animals were euthanized 48 hours post infection. Serum and tissue samples were analyzed for bacterial colony counts, cytokine levels, splenocyte apoptosis rates and cell population analysis by flow cytometry. In parallel, specific miRNA expression analyses in splenocytes and lung histologic examination were also performed. Comparisons of numeric data between groups were made using the one-way ANOVA test for multiple groups.

## Results

We found that upon NK cell depletion there was a significant increase in the spleen NKT (CD3^+^/CD1d^+^) cell population compared with NKT^DEPL^, C and S (*P *= 0.014, *P *= 0.021 and *P *= 0.033, respectively). Interestingly, upon NKT cell depletion, spleen NK (CD3^-^/NK1.1^+^) cells increased significantly compared with NK^DEPL^, C and S (*P *< 0.0001, *P *< 0.0001 and *P *= 0.001, respectively). NKT depletion led to decreased lymphocyte apoptosis compared with C (*P *= 0.035), higher bacterial load in the lung compared with C and NK^DEPL ^(*P *= 0.014 and *P *= 0.022 respectively) and in the liver compared with C (*P *= 0.012). In addition, serum levels of IFNγ were significantly increased and splenocytes from NKT depleted animals, incubated *ex vivo *in the presence or absence of IL-2, produced more IFNγ in comparison with all other groups. Furthermore, splenocyte miRNA analysis showed that miR-200c and miR-29a were downregulated, while miR-125a-5p was upregulated, in the NKT depleted animals compared with all other groups.

## Conclusion

For the first time we have shown that NKT cell depletion resulted in an increase in spleen NK (CD3^-^/NK1.1^+^) cells and a higher IFNγ production, which were associated with specific changes in splenocyte miRNA expression.

